# Confirmation of ovulation from urinary progesterone analysis: assessment of two automated assay platforms

**DOI:** 10.1038/s41598-018-36051-6

**Published:** 2018-12-04

**Authors:** Robert M. Gifford, Forbes Howie, Kirsten Wilson, Neil Johnston, Tommaso Todisco, Mike Crane, Julie P. Greeves, Karolina Skorupskaite, David R. Woods, Rebecca M. Reynolds, Richard A. Anderson

**Affiliations:** 1 University/British Heart Foundation Centre for Cardiovascular Science, Queen’s Medical Research Institute, University of Edinburgh, Edinburgh, EH16 4TJ UK; 2 Research & Clinical Innovation, Royal Centre for Defence Medicine, Birmingham, UK; 3 MRC Centre for Reproductive Health, Queen’s Medical Research Institute, University of Edinburgh, Edinburgh, UK; 4 Department of Biochemistry, Royal Infirmary of Edinburgh and Royal Hospital for Sick Children, NHS Lothian, Edinburgh, UK; 5 Army Personnel Research Capability, Andover, UK; 6 Research Institute for Sport, Physical Activity and Leisure, Leeds Beckett University, Leeds, UK; 7 Northumbria and Newcastle NHS Trusts, Wansbeck General and Royal Victoria Infirmary, Newcastle, UK; 8 University of Newcastle, Newcastle upon Tyne, UK

## Abstract

Urinary concentrations of the major progesterone (P4) metabolite pregnanediol-3-glucuronide (PDG) are used to confirm ovulation. We aimed to determine whether automated immunoassay of urinary P4 was as efficacious as PDG to confirm ovulation. Daily urine samples from 20 cycles in 14 healthy women in whom ovulation was dated by ultrasound, and serial weekly samples from 21 women in whom ovulation was unknown were analysed. Daily samples were assayed by two automated P4 immunoassays (Roche Cobas and Abbott Architect) and PDG ELISA. Serial samples were assayed for P4 by Architect and PDG by ELISA. In women with detailed monitoring of ovulation, median (95% CI) luteal phase increase was greatest for PDG, 427% (261–661), 278% (187–354) for P4 Architect and least for P4 Cobas, 146% (130–191), p < 0.0001. Cobas P4 also showed marked inaccuracy in serial dilution. Similar ROC AUCs were observed for individual threshold values and two-sample percent rise analyses for P4 Architect and PDG (both >0.92). In serial samples classified as (an)ovulatory by PDG, P4 Architect gave ROC AUC 0.95 (95% CI 0.89 to 1.01), with sensitivity and specificity for confirmation of ovulation of 0.90 and 0.91 at a cutoff of 1.67 μmol/mol. Automated P4 may potentially be as efficacious as PDG ELISA but research from a range of clinical settings is required.

## Introduction

The confirmation of ovulation is important for the investigation of infertility, for women planning conception and for researchers understanding the impact of interventions on ovarian function. Transvaginal ultrasound (TVUS) detection of the growth and disappearance of a follicle is the gold standard technique, but it is invasive and repeated measures are often undesirable or not feasible in an outpatient setting^1,2^. The assessment of cyclical hormonal concentrations represents an objective alternative. Clinical guidelines suggest blood measurement of progesterone (P4) and gonadotrophins during the luteal phase^3–5^. Elevated serum P4 has high specificity, but may require repeated venipuncture and is invasive^6^. The use of assays of urinary metabolites of P4 (pregnanediol 3 glucuronide, PDG) and oestradiol (e.g. estrone-3-glucuronide) and/or luteinizing hormone (LH), correcting for urinary creatinine to adjust for an individual’s fluid status, has been widely used for many decades and allows convenient repeated sample collection^7,8^. P4 is inactivated to pregnanediol by reduction at the C5, C3 and C20 position, and glucuronic acid is attached via a glycosidic bond, forming PDG.

A rise in urine PDG above a certain threshold (commonly 5 μg/mL) is required to confirm ovulation^9^. Urine PDG demonstrates excellent agreement with P4 in both serum^10,11^ and urine^12^. In practice, PDG assays often require a relatively onerous manual competitive ELISA, and the related expense limits the availability of testing in a clinical setting. Unlike PDG, assays for P4 are readily available for automated analyzers commonly used in hospital laboratories, and therefore have potential advantages in expediency, cost-effectiveness and availability.

We therefore aimed to determine whether daily measurement of creatinine-corrected urinary progesterone using an automated progesterone assay could be used for reliable confirmation of ovulation in a cohort in whom ovulation had already been reliably identified (confirmatory cohort). We also aimed to explore whether a P4 threshold value for one sample, or a percent rise between two samples (one follicular and one luteal) was the more discriminatory in the confirmatory cohort. Furthermore, in weekly samples from an additional cohort, in whom no further information on ovulation status was known (exploratory cohort), we aimed to explore the sensitivity and specificity of weekly P4 in confirming ovulation (threshold value and two sample percent rise) with PDG as the referent.

## Materials and Methods

### Human subject recruitment

Ethical approval was obtained from South East Scotland Research Ethics Committee (Ref: 09/S1101/67). The study conformed to the principles outlined in the Declaration of Helsinki. The study consisted of two cohorts: a confirmatory cohort, evaluating the ability of urine P4 to confirm ovulation diagnosed by ultrasound, relative to PDG, and an exploratory cohort, comparing P4 and PDG in a likely real-world setting, where true ovulation status is unknown, daily testing is not possible and weekly urinary PDG is used clinically.

The confirmatory cohort comprised 14 healthy women aged 27 to 43 years with self-reported regular menses, who were controls in a study in which timing of ovulation was characterized using gold-standard techniques^13^. All provided informed consent. Inclusion criteria were: reproductive age (between menarche and menopause), no steroidal contraception or other hormonal medication, intrauterine device use or fertility treatment, normal physical examination, and renal and liver function and electrolytes within normal limits. Participants were excluded if they could not complete the required sampling regimen or became pregnant.

The exploratory cohort comprised 21 women attending a reproductive endocrinology service in Edinburgh, undergoing a standard clinical assessment. None were taking hormonal contraceptives or fertility treatment, all were of reproductive age and all had completed the required sampling regimen. Informed consent was not required for the exploratory cohort, since the investigation was part of their routine investigations. Investigators were blinded to the findings of history, examination and other investigations. Seven healthy men aged 28 to 61 years provided single urine aliquots for assessment of linearity and dilution recovery.

### Sample size

Since confirmatory and exploratory cohorts were assessed as part of other research or clinical activities, they may be considered convenience samples. Although sample sizes (confirmatory: 20 cycles from 14 women, exploratory: 42 cycles from 21 women) were smaller than previous studies^1,11,14–16^, assessments were in greater detail (e.g. daily and weekly urine sampling, respectively, see ‘*Capability in confirming ovulation’*), hence we anticipated the sample size would be sufficient to confirm the ability of urinary P4 to identify ovulation and explore its diagnostic utility versus PDG.

### Creatinine and LH assays

LH was measured in serum and urine by in-house ELISA using two different anti-human LH beta subunit mouse monoclonal antibodies (Medix Biochemica, Kauniainen, Finland), as described elsewhere^17^. While LH may be unstable in urine at −20 °C^18^, a measured peak in urine LH would intended to be supportive of a serum measurement, and TVUS, both within 2–3 days. Urine creatinine was determined using the creatininase/creatinase specific enzymatic method utilizing a commercial kit (Alpha Laboratories Ltd. Eastleigh, UK) adapted for use on a Cobas Fara centrifugal analyser (Roche Diagnostics Ltd, Welwyn Garden City, UK)^19^.

### Urine steroid assays

Urine samples were stored at −20 °C until steroid analysis. Measurement of PDG and two automated P4 immunoassays were undertaken on each sample.

PDG was measured in duplicate by competitive PDG ELISA. A 96-well plate was coated with 100 μL of pre-precipitated donkey 0.2 μg anti rabbit IgG serum per well (Scottish Antibody Production Unit, Carluke, UK) in ELISA coating buffer for 14 hours at 4 °C and washed twice with 50 mM TRIS buffer containing 137 mM NaCl and 0.05% tween 20 (wash buffer). The plate was blocked with 220 μL 10 mM phosphate buffer and 0.5% w/v bovine serum albumin (BSA), washed twice with wash buffer, 20 μL of sample was added with 80 μL PDG-HRP (in house reagent) 1 in 200,000 in PBS 0.1%BSA (assay buffer) and shaken for 2 minutes. 50 μL Rabbit anti-PDG Ab (in house reagent) 1 in 40,000 assay buffer were then added and incubated in a shaker at 30 °C for 2 h. The plates were washed 5 times with wash buffer and 120 μL 3,3′,5,5′-Tetramethylbenzidine (TMB) was added. After 12–15 minutes 80 μL 2 M H_2_SO4 stop solution was added and the plate read on a plate reader at 450 nm.

Automated P4 chemiluminescent microparticle immunoassay (Abbott Laboratories, Lake Bluff, Illinois, USA): P4 was measured on the Abbott Architect c8000 automated analyser, using a proprietary serum assay kit (Architect System Progesterone, Abbott Ireland Diagnostics Division, Longford, Ireland) according to the manufacturer’s instructions. The analytical sensitivity was quoted as ≤0.3 nmol/L. No significant cross-reactants are quoted by the manufacturer.

Automated P4 electrochemiluminescence immunoassay (Roche Diagnostics Ltd, Welwyn Garden City, UK): P4 was measured on the Roche Cobas automated immunoanalyser, using a proprietary serum assay kit (Elecsys® Progesterone II, Roche Diagnostics, Indianapolis, Indiana, USA) according to the manufacturer’s instructions. The analytical sensitivity quoted as 1.0 nmol/L. The only significant cross-reactant quoted by the manufacturer is 5 β-dihydroprogesterone at 20.7%.

### Comparison and correlation between assay methods

A total of 536 daily, early morning urine samples were assayed using PDG ELISA, Architect and Cobas P4.

### Cross-reactivity of P4 asssays for PDG

Since neither P4 assay quoted cross-reactivity with PDG, we measured P4 using both assays in three spiked male urine samples (100 nM PDG) and three unspiked samples. Mean progesterone concentrations were compared between spiked and unspiked samples to give percent crossreactivity.

### Freeze thaw stability

There was one freeze-thaw cycle between each assay, in the order PDG, Cobas, Architect. PDG is known to be stable following up to 10 freeze-thaw cycles^11^, but the stability of urinary progesterone measured by automated assay has not previously been demonstrated. We examined the effect of up to five freeze-thaw cycles on hormone concentration. An aliquot of male urine was spiked with 240nmol/L P4 and divided into 18 aliquots. Three aliquots were each subjected to 0, 1, 2, 3, 4 or 5 freeze-thaw cycles. P4 was then measured using both assays and the percentage decrease from the index samples (0) calculated.

### Assay precision

Standard samples supplied by the manufacturers spanning the low, middle and high range were measured with both P4 methods at four different time points within each run (Architect 4 runs, Cobas 5 runs). Runs were carried out on separate days and the reagent lot was varied to simulate normal operating procedures.

Within-assay precision was determined by repeating the assays in four replicates simultaneously for creatinine, LH and PDG. The intra-assay CVs were <3%, <5% and <10% while inter-assay CVs were <5%, <10% and <10% for creatinine, LH and PDG, respectively.

### Linearity and dilution recovery

Seven male samples were spiked with 12.5 mmol/L P4 and were diluted serially with unspiked male urine 2, 4 and 8-fold. These samples were tested on both P4 methods, to assess if any disparity diminished with subsequent dilutions.

### Capability in confirming ovulation

Daily early morning urine samples were collected across 20 menstrual cycles from the confirmatory cohort. Ovulation was confirmed by the appearance and disappearance of a dominant follicle on transvaginal ultrasound (TVUS), performed every 2–3 days. The precise day of ovulation was determined from the surge in daily urinary LH, corroborated by serum LH (measured every 2–3 days). Samples from ovulation day −10 to −3 were categorized as follicular, and ovulation day +3 to +10 as luteal.

In order to compare the rates of confirmation of ovulation for P4 Architect with PDG ELISA, a further eight, weekly samples were assayed from the confirmatory cohort (in whom the presence or absence of ovulation was otherwise undetermined). These women provided one urine sample every seven days for eight weeks, starting on a random day of the cycle.

### Statistical Analysis

Non-normally distributed data were log transformed prior to analysis. Correlation of P4 assays with PDG was performed by Pearson’s correlation analysis. Values obtained by Cobas and Architect were also compared using Passing-Bablok regression. A Bland-Altman plot was used to check graphically for systematic bias and heterogeneity across the range of values. For assay precision, a coefficient of variance (CV) within or between assays of 10% or less was considered acceptable. We estimated within and between series imprecision using analysis of variance (ANOVA). For linearity and dilution recovery, the correlation between observed and expected values was compared using Pearson’s test.

To assess the performance in confirming ovulation, all three assays were compared graphically by plotting the median, 10^th^ and 90^th^ percentile concentrations by day of ovulation. Follicular and luteal P4 concentrations by each assay were compared using paired samples t tests. Percent luteal change for all three assays was calculated using the median ratio of all combinations of follicular and luteal creatinine-corrected concentrations by one-way ANOVA. The sensitivity and specificity of PDG was compared with the closest-correlating P4 assay using 1. Daily urine samples (ovulation confirmed above a threshold concentration) and 2. A percent-rise between pairs of samples (ovulation confirmed above a certain percent rise) to confirm ovulation. Receiver-operator characteristics (ROC) curves were constructed and compared.

To compare the ovulation detection rate of P4 with P3G in the exploratory cohort, the diagnostic threshold concentrations and ratios calculated by the ROC curves in the confirmatory cohort were applied to these weekly samples. Where PDG exceeded the threshold concentration, this cycle was deemed ovulatory. The rise in PDG or P4 was assigned as week 3 for graphical purposes. Other cycles were deemed anovulatory. Luteal percentage changes were calculated as the difference between a sample and each of the other seven weekly samples (56 combinations for each woman). The sensitivity and specificity of P4 relative to PDG (the referent) were calculated for anovulatory and ovulatory cycles. The peak samples from each 4-sample consecutive series were analysed by ROC curve.

A p-value < 0.05 was considered statistically significant. Statistical analyses were undertaken using Analyse-It version 2.2 (Leeds, UK) and SPSS Statistics for Mac version 23.0 (IBM, New York, USA).

## Results

### Comparison and correlation between assay methods

**Figure 1:**
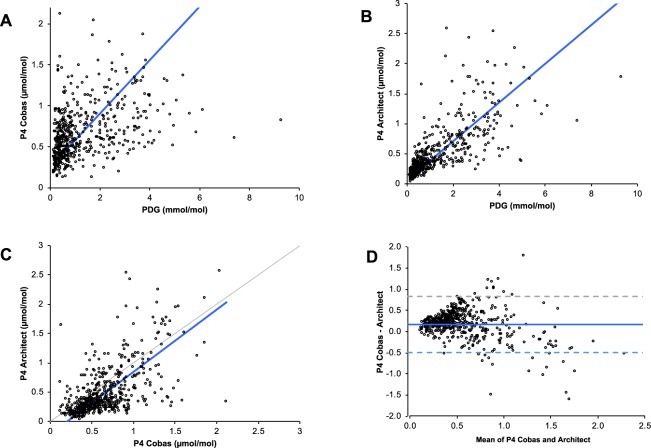
Passing Bablok plot of creatinine-corrected P4 Cobas and PDG ELISA (top left panel), P4 Architect and PDG ELISA (top right panel), P4 Architect and P4 Cobas (bottom left panel) and Bland Altman Plot of the two P4 assays (bottom right panel). P4: Progesterone, PDG: pregnanediol glucuronide, Cobas: Roche Cobas e411 electrochemiluminescence assay, Architect: Abbott Architect chemiluminescent microparticle immunoassay.

Bland-Altman analysis demonstrated positive bias towards Cobas values with a trend towards greater discrepancy at lower concentrations of P4 than by Architect (Fig. 1). Passing Bablok plots similarly demonstrated higher values by Cobas at lower concentrations, with higher values by Architect at higher concentrations. The correlations between PDG and P4 by Cobas and P4 by Architect were r = 0.454, r = 0.708, respectively, both p < 0.0001.

### Cross-reactivity of P4 asssays for PDG

The cross-reactivity for PDG in urine was 0.59% for Architect and 0.54% for Cobas.

### Freeze-thaw stability of P4

The mean (SD) concentration of the index samples was Architect: 247 (3.52) nmol/L, Cobas: 249 (6.3) nmol/L. The percent change in P4 following freeze thaw cycles is shown in Supplementary Table 1. After 1 freeze-thaw cycle, the mean (SD) change in concentration for Architect and Cobas, respectively was −2.61 (0.93) % and −2.21 (6.01) %, after 3 freeze thaw cycles −4.85 (2.99) % and +3.30 (7.12) %, and after 5 freeze-thaw cycles −7.60 (3.23) % and −7.02 (2.99) %.

### Assay precision

Coefficient variations for Architect and Cobas were within-run: < 2.5% and < 0.8%, and between-run: <3.8% and <1.4%, respectively.

### Linearity and dilution recovery

P4 recovery measured by Architect demonstrated high linearity and close to 100% recovery, whereas Cobas demonstrated excess recovery (Table 1). The disparity between observed and expected values by Cobas diminished following serial dilution (170% spiked undiluted and 143% following 8x dilution, versus 113% and 112%, for Architect respectively), indicating a likely matrix effect. Pearson’s correlations with expected values were r = 0.987 for Architect and r = 0.947 Cobas.

**Table 1:** Analytical recovery of P4 from urine in the 2 automated assays. SD; standard deviation. Architect: Abbott Architect progesterone chemiluminescent microparticle immunoassay, Cobas: Roche Cobas electrochemiluminescence immunoassay, P4: progesterone.

P4 added, nmol/L	% recovery, mean (SD)	
	Cobas	Architect
**Male**		
12.5	170 (41.7)	113 (13.3)
6.25 (x2 dilution)	160 (30.0)	106 (16.4)
3.13 (x4 dilution)	153 (26.6)	109 (16.7)
1.56 (x8 dilution)	143 (22.0)	112 (67.0)

N = 7.

### Capability in confirming ovulation

**Figure 2:**
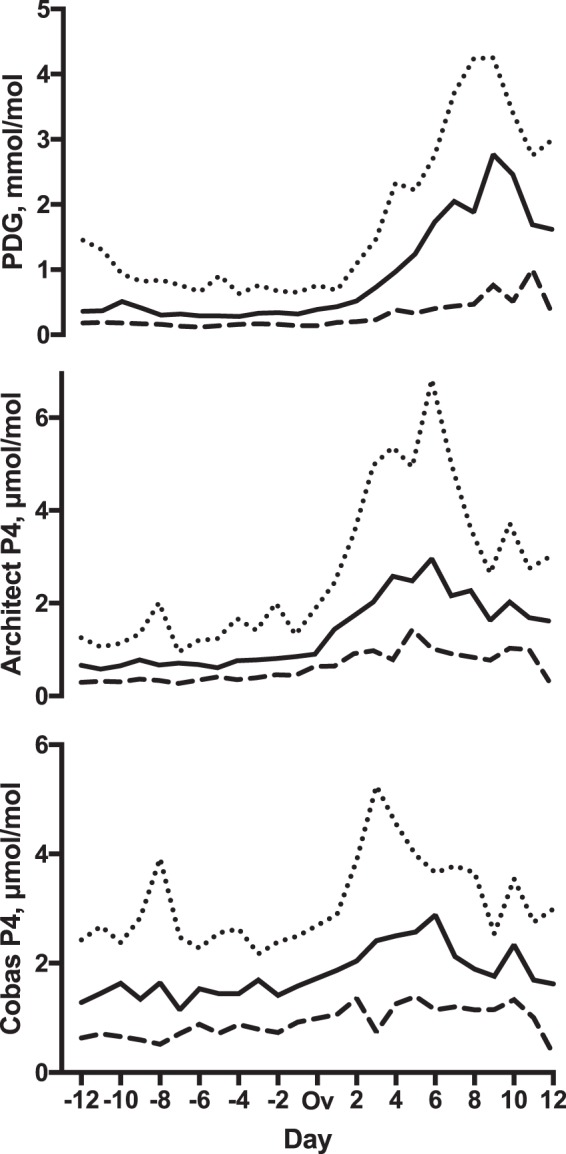
Analysis of pregnanediol glucuronide (PDG) and progesterone (P4) in daily uring samples in the confirmatory cohort (ovulation identified by ultrasound). Solid line – median. Dashed line – 10^th^ centile. Dotted line – 90^th^ centile. Ov: day of ovulation. Day represents cycle day relative to day of ovulation. PDG: pregnanediol-3-glucuronide, P4: Progesterone, Architect: Abbott Architect chemiluminescent microparticle immunoassay. Cobas: Roche Cobas e411 electrochemiluminescence immunoassay.

**Figure 3:**
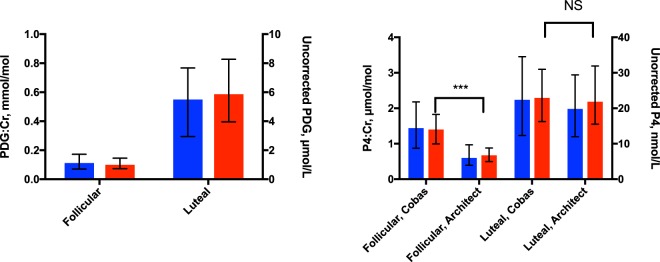
Median, luteal and follicular values, uncorrected and corrected for creatinine. Median, luteal and follicular values, uncorrected (blue) and corrected for creatinine (red). Values are median (IQR). PDG:Cr, pregnanediol glucuronide corrected for creatinine, P4:Cr progesterone, corrected for creatinine; Architect: Abbott Architect progesterone chemiluminescent microparticle immunoassay. Cobas: Roche Cobas progesterone electrochemiluminescence immunoassay ***p < 0.0001 NS p > 0.05.

In the confirmatory cohort, the median (range) cycle length was 28 (25–38) days. Day of ovulation was confirmed by transvaginal ultrasound and serum and urinary LH peak (median day 14 range day 12 – day 20). The median, 10^th^ and 90^th^ centiles of corrected PDG and P4 measured by Cobas and Architect by cycle day are illustrated in Fig. 2. Follicular P4 was higher when measured by Cobas than by Architect (median (IQR) creatinine-corrected follicular concentration 1.41 (1.14–1.85) nmol/mol versus 0.66 (0.50–0.89) nmol/mol, respectively, p < 0.0001), while luteal concentrations did not significantly differ (2.30 (1.63–3.13) nmol/mol versus 2.19 (1.52–3.24) nmol/mmol, respectively, p = 0.7) (Fig. 3). The median (IQR) percent luteal change for Cobas P4, Architect P4 and PDG were 146 (130–191)%, 278 (187–354)%, and 427 (261–661)%, respectively, p < 0.0001.

**Figure 4:**
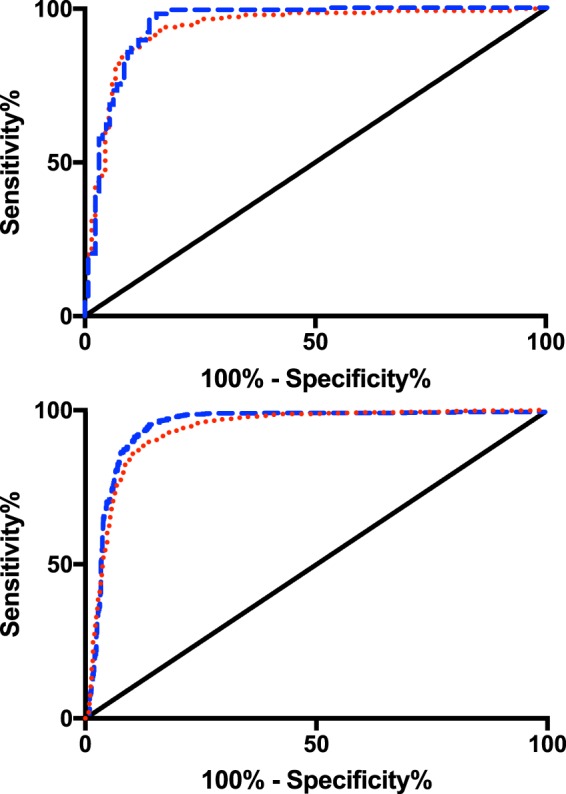
Receiver-Operator Characteristics creatinine-corrected PDG ELISA versus creatinine-corrected Architect P4 for single-sample threshold concentration (top panel) and two-sample follicular to luteal rise (bottom panel). Top panel: single sample threshold concentration to confirm ovulation; area under curve (95% CI) for P4 threshold (red dotted line) was 0.944 (0.916–0.973) and for PDG threshold (blue dashed line) was 0.951 (95% CI 0.923-0.978), p = 0.7. Bottom panel: two-sample luteal rise to confirm ovulation. Area under the curve (95% CI) for P4 two-sample difference (red dotted line) was 0.927 (0.915–0.940) and for PDG two-sample difference (blue dashed line) 0.950 (0.940–0.961), p = 0.003. Solid line: identity.

The Architect P4 assay was therefore chosen for comparison with PDG for efficacy in confirming ovulation. Assay ROC areas under curve (AUCs) for single sample threshold were 0.951 (95% CI 0.923 to 0.978) and 0.944 (95% CI 0.916 to 0.973), for PDG and P4 respectively (p = 0.7). The ROC AUCs for luteal percent rise were 0.927 (95% CI (0.915 to 0.940) and 0.950 (95% CI (0.940 to 0.961), respectively (p = 0.003) (Fig. 4). The optimal individual sample threshold identified to confirm ovulation for P4 and PDG were 1.14 μmol/mmol and 0.208 mmol/mmol, respectively, yielding sensitivity and specificity of 0.88 to 0.99 with no meaningful differences between single threshold concentration or luteal percent rise, or between assays (Table 2). There was no significant difference between P4 and PDG ROC AUCs for threshold concentration (Table 3). The optimal percent luteal value rise for P4 and PDG were 165% and 195%, respectively.

**Table 2:** Sensitivity specificity and predictive values of corrected pregnanediol and P4 by Architect assay single values and two-sample luteal percent increase (confirmatory cohort - ovulation identified by ultrasound). PDG, pregnanediol glucuronide. P4, progesterone.

Indicator of ovulation	Value	Sensitivity	Specificity	Positive predictive value	Negative predictive value
PDG follicular to luteal percent-rise, %	**195**	0.90	0.90	0.90	0.90
P4 follicular to luteal percent-rise, %	**165**	0.88	0.88	0.87	0.88
PDG threshold value, mmol/mol	**1.14**	0.89	0.90	0.88	0.91
P4 luteal threshold value, μmol/mol	**0.208**	0.88	0.90	0.89	0.90

**Table 3:** Differences between areas under receiver operator characteristics curves between progesterone (P4) and pregnanediol (PDG) single-sample threshold and two-sample percent rise to confirm ovulation (difference and non-directional p value (two-tailed)), in cohort of women in whom ovulation had been confirmed. PDG, pregnanediol glucuronide; P4 progesterone; CI, confidence interval.

	P4 single value 0.944 (95% CI 0.916–0.973	P4 luteal percent increase 0.927 (95% CI (0.915 – 0.940)	PDG luteal 0.951 (95% CI.923–0.978)	PDG luteal percent increase 0.950 (95% CI (0.940–0.961)
P4 single value 0.944 (95% CI 0.916–0.973		Difference 0.017 P = 0.278	Difference 0.007 P = 0.730	Difference 0.006 P = 0.696
P4 luteal percent increase 0.927 (95% CI (0.915 – 0.940)	Difference –0.017 P = 0.278		Difference 0.0151 P = 0.112	Difference 0.023 P = 0.003
PDG single value 0.951(95% CI.923–0.978)	Difference –0.007 P = 0.730	Difference –0.0151 P = 0.112		Difference 0.001 P = 0.946
PDG luteal percent increase 0.950 (95% CI (0.940–0.961)	Difference –0.006 P = 0.696	Difference –0.023 P = 0.003	Difference –0.001 P = 0.946	

**Figure 5:**
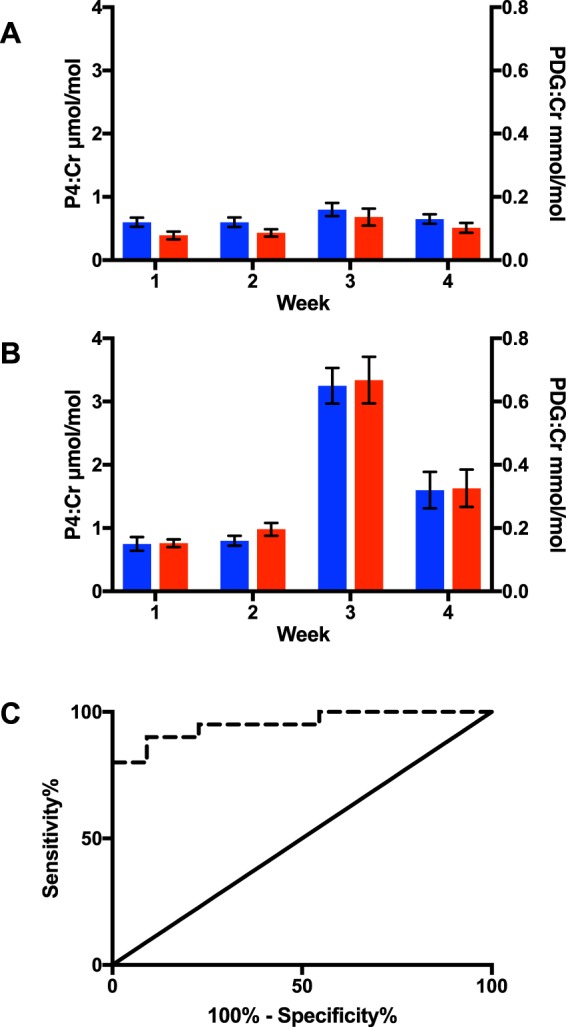
Weekly creatinine-corrected PDG and P4 in the exploratory cohort. (**A**) anovulatory cycles (n = 22, 14 women), (**B**) ovulatory cycles (n = 20, 13 women). Values are mean ± SEM. Ovulation was determined by reaching a threshold cutoff in PDG and week 3 designated by the rise in PDG/P4. C:Receiver operator characteristics (ROC) for peak P4:Cr from each 4-sample consecutive series. Area under the curve 0.952 (95% CI 0.89 to 1.01). Blue bars, PDG:Cr: urinary creatinine-corrected pregnanediol glucuronide, red bars, P4:Cr: urinary creatinine-corrected progesterone. Dashed line: ROC, solid line: identity.

In the exploratory cohort, 20 ovulatory and 22 anovulatory cycles were identified (Fig. 5A,B). For ovulatory cycles (n = 20, from 13 women), sensitivity and specificity of P4 compared with PDG were threshold: 0.89 and 0.95, percent change: 0.95 and 0.91, respectively. For anovulatory cycles (n = 22, from 14 women), sensitivity and specificity of P4 compared with PDG were threshold: 1.00 and 0.98, percent change: 0.87 and 0.92 (Table 4). Receiver-operator characteristic AUC for peak values identified from ovulatory and anovulatory women was 0.95 (95% CI 0.89 to 1.01). At a cut-off P4 of 1.67 μmol/mol, sensitivity was 0.90 and specificity 0.91.

**Table 4:** Comparison of progesterone (P4) sensitivity and specificity relative to pregnanediol glucuronide (PDG) in the exploratory cohort (ovulation had not been otherwise identified). P4, progesterone; PDG, pregnanediol glucuronide.

	PDG above luteal threshold concentration	PDG below luteal threshold conentration	
Ovulatory cycles (n = 20, from 13 women)			
P4 single sample cut-off confirmed ovulation	25	2	Sensitivity of single sample cut-off P4 relative to PDG: 0.89
P4 single sample cut-off did not confirm ovulation	3	41	Specificity of single sample cut-off P4 relative to PDG: 0.95
P4 two sample percent rise confirmed ovulation	139	38	Sensitivity of P4 two sample percent rise relative to PDG: 0.95
P4 two sample percent rise did not confirm ovulation	8	375	Specificity of P4 two sample percent rise relative to PDG: 0.91
Anovulatory cycles (n = 22, from 14 women)			
P4 single sample cut-off confirmed ovulation	0	2	Sensitivity of single sample cut-off P4 relative to PDG: 1.00
P4 single sample cut-off did not confirm ovulation	0	86	Specificity of single sample cut-off P4 relative to PDG: 0.98
P4 two sample percent rise confirmed ovulation	26	47	Sensitivity of P4 two sample percent rise relative to PDG: 0.87
P4 two sample percent rise did not confirm ovulation	4	539	Specificity of P4 two sample percent rise relative to PDG: 0.92

## Discussion

In a sample of women in whom ovulation was carefully characterized (confirmatory cohort) we found that measurement of urinary P4 using an automated assay reproducibly demonstrated comparable relative concentration changes to PDG. Compared with PDG, the Architect P4 assay demonstrated satisfactory receiver operator characteristics and positive and negative predictive values.

In women in whom ovulation was otherwise undefined (exploratory cohort), P4 Architect was closely comparable to PDG concentration as the referent, with AUC CIs crossing unity and sensitivity and specificity of 90% and 91% respectively. The luteal percent change method estimated a marginally lower sensitivity than single threshold concentration, however this was likely due to the impartial analytical approach (each weekly sample was compared with seven other samples). We were unable to demonstrate any difference between a single threshold value or percent luteal rise in confirmation of likely ovulation.

The Architect method demonstrated a greater luteal rise for P4 than reported by Stanczyk *et al*.^12^, however this was still significantly less than was seen for PDG. A specific ELISA for PDG remains superior in confirming ovulation using urine samples to either automated P4 assay. In the exploratory cohort, the sensitivity and specificity of P4 were calculated relative to PDG, as a gold-standard technique such as TVUS had not been undertaken. This confirmed the potential of urinary P4 analysis using the Architect system for confirmation of ovulation in a clinical setting. The manual PDG assay however requires several hours to perform with overnight plate coating and/or antibody incubation^11,20^. Such a time investment will carry cost implications. Recent studies advocating the use of a PDG threshold concentration to confirm ovulation utilized a time-resolved fluorimetric immunosorbent assay, but details of the assay were not described^9,10,14,15^. Time-resolved fluorimetry requires more specialised equipment than the competitive TMB-based ELISA, hence it is likely this technique will be predominantly utilized by specialised reproductive laboratories. Liquid chromatography and tandem mass spectroscopy represent an accurate alternative^21^, however the cost is likely to be prohibitive in the general laboratory. Autoanalyzers such as those tested here are in widespread use for plasma/serum in general biochemistry laboratories and using them for confirmation of ovulation, where available, would be of great practical value, reducing direct and indirect costs and improving efficiency. The message is autoanalyser use improves efficacy and is practical so is less resource intensive than a manual ELISA. They are also less likely to be associated with human error. The Abbott and Cobas P4 assays are not developed or marketed for urine but this analysis suggests that the Abbott assay shows good characteristics and may potentially be of clinical value in this context, after further validation in larger cohorts. While Architect P4 demonstrated a correlation with PDG of r = 0.71, it shows potential for clinical application, since the identification of change from follicular to luteal concentrations is robust (90% sensitive and 91% specific in this exploratory cohort of 21 women). It may also prove a useful tool for population-based research studies, where large numbers of samples need to be analysed.

The Cobas demonstrated a matrix effect for measurement of P4 in urine, overestimating concentrations thus limiting the assay’s ability to differentiate between follicular and luteal samples. Cobas also demonstrated a poor percentage recovery, an effect which was reduced by serial dilutions with phosphate buffered saline. Architect by comparison was unaffected by matrix effect in urine and showed good recovery, and thus was chosen for further comparisons.

As far as we are aware this is the first time PDG ELISA has been compared with an automated assay of P4 in urine for the confirmation of ovulation. Strengths of our study include the detailed assessment of ovulation and excellent adherence to a daily urine sampling regimen. Our study has several weaknesses. An important limitation is that the confirmatory population was relatively small, although statistical significance was achieved for the key comparison of fold increase in luteal versus follicular P4. Ultrasound, blood and urine hormone measurements in this cohort provide more detail than previous studies and represent a gold standard of ovulation determination, hence we feel this sample size was sufficient to confirm the ability of daily urinary Architect P4 to identify ovulation. Nevertheless these data should therefore be interpreted with caution and substantially larger sample sizes are required for determination of reference ranges for threshold or cutoff values. Our assays were not contemporaneous, with 1 freeze-thaw cycle between each of them. While significant degradation of steroids was not detected, in future researchers should aim to run the assays concurrently^11,22^.

Larger studies including TVUS in both ovulatory and anovulatory women are required to determine the best sampling strategy to confirm ovulation. It would also be necessary to determine the efficacy of automated P4 assessment versus PDG in a range of ovulatory patterns before recommending this test for widespread clinical use, for which sufficient reliability has not yet been demonstrated.

## Electronic supplementary material

Supplementary table 1

## Data Availability

The datasets generated and analysed during the current study are available from the corresponding author.

## References

[1] Lynch KE (2014). Assessment of anovulation in eumenorrheic women: comparison of ovulation detection algorithms. Fertility and Sterility.

[2] Guermandi E (2001). Reliability of ovulation tests in infertile women. Obstetrics and gynecology.

[3] NICE. Clinical guideline [CG156] Fertility problems: assessment and treatment. Section 1.3.4 (2017).

[4] Kamel RM (2010). Management of the infertile couple: an evidence-based protocol. Reprod Biol Endocrinol.

[5] Lindsay TJ, Vitrikas KR (2015). Evaluation and treatment of infertility. Am Fam Physician.

[6] Su H-W, Yi Y-C, Wei T-Y, Chang T-C, Cheng C-M (2017). Detection of ovulation, a review of currently available methods. Bioengineering & Translational Medicine.

[7] Baird DD, Weinberg CR, Wilcox AJ, McConnaughey DR, Musey PI (1991). Using the ratio of urinary oestrogen and progesterone metabolites to estimate day of ovulation. Statistics in medicine.

[8] Blackwell LF, Cooke DG, Brown S (2018). The Use of Estrone-3-Glucuronide and Pregnanediol-3-Glucuronide Excretion Rates to Navigate the Continuum of Ovarian Activity. Frontiers in Public Health.

[9] Ecochard R (2013). Use of urinary pregnanediol 3-glucuronide to confirm ovulation. Steroids.

[10] Roos J (2015). Monitoring the menstrual cycle: Comparison of urinary and serum reproductive hormones referenced to true ovulation. The European Journal of Contraception & Reproductive Health Care.

[11] O’Connor KA (2003). Urinary Estrone Conjugate and Pregnanediol 3-Glucuronide Enzyme Immunoassays for Population Research. Clinical chemistry.

[12] Stanczyk FZ (1997). Urinary progesterone and pregnanediol. Use for monitoring progesterone treatment. The Journal of reproductive medicine.

[13] Skorupskaite K, George JT, Veldhuis JD, Anderson RA (2017). Neurokinin B regulates gonadotropin secretion, ovarian follicle growth and the timing of ovulation in healthy women. The Journal of clinical endocrinology and metabolism.

[14] Ecochard R (2017). Characterization of hormonal profiles during the luteal phase in regularly menstruating women. Fertil Steril.

[15] Leiva R, Bouchard T, Boehringer H, Abulla S, Ecochard R (2015). Random serum progesterone threshold to confirm ovulation. Steroids.

[16] Abdulla SH (2018). Hormonal Predictors of Abnormal Luteal Phases in Normally Cycling Women. Front Public Health.

[17] George JT (2011). Kisspeptin-10 is a potent stimulator of LH and increases pulse frequency in men. The Journal of clinical endocrinology and metabolism.

[18] Robinson N, Saudan C, Sottas P-E, Mangin P, Saugy M (2007). Performance characteristics of two immunoassays for the measurement of urinary luteinizing hormone. Journal of pharmaceutical and biomedical analysis.

[19] Borner U, Szasz G, Bablok W, Busch EW (1979). [A specific fully enzymatic method for creatinine: reference values in serum (author’s transl)]. *Journal of clinical chemistry and clinical biochemistry*. Zeitschrift fur klinische Chemie und klinische Biochemie.

[20] Munro CJ (1991). Relationship of serum estradiol and progesterone concentrations to the excretion profiles of their major urinary metabolites as measured by enzyme immunoassay and radioimmunoassay. Clinical chemistry.

[21] Sinreih M (2015). Combined liquid chromatography-tandem mass spectrometry analysis of progesterone metabolites. PLoS One.

[22] Reyna R, Traynor KD, Hines G, Boots LR, Azziz R (2001). Repeated freezing and thawing does not generally alter assay results for several commonly studied reproductive hormones. Fertil Steril.

