# Supplementary material for: Confirmation of ovulation from urinary progesterone analysis: assessment of two automated assay platforms
Source: Sci Rep. 2018 Dec 4;8:17621. doi: 10.1038/s41598-018-36051-6 (PMC6279762; doi:10.1038/s41598-018-36051-6)
Supplement: Supplementary file 1 — Supplementary table 1 [file 41598_2018_36051_MOESM1_ESM.pdf]

Supplementary Information

Main Article title: Confirmation of ovulation from urinary progesterone analysis: assessment of two automated assay platforms

Robert M Gifford [1,2], Forbes Howie [3], Kirsten Wilson [3], Neil Johnston [1], Tommaso Todisco [1], Mike Crane [4], Julie P Greeves,[5] Karolina Skorupskaite [3], David R Woods [2,6,7,8], Rebecca M Reynolds [1], Richard A Anderson [3]

1 University/British Heart Foundation Centre for Cardiovascular Science, Queen’s Medical Research Institute, University of Edinburgh, Edinburgh, UK EH16 4TJ.
2 Research & Clinical Innovation, Royal Centre for Defence Medicine, Birmingham, UK
3 MRC Centre for Reproductive Health, Queen's Medical Research Institute, University of Edinburgh, Edinburgh, UK
4 Department of Biochemistry, Royal Infirmary of Edinburgh and Royal Hospital for Sick Children, NHS Lothian, Edinburgh, UK
5 Army Personnel Research Capability, Andover, UK
6 Research Institute for Sport, Physical Activity and Leisure, Leeds Beckett University, Leeds, UK
7 Northumbria and Newcastle NHS Trusts, Wansbeck General and Royal Victoria Infirmary, Newcastle, UK
8 University of Newcastle, Newcastle upon Tyne, UK

Corresponding Author: Dr Robert M Gifford, Centre for Cardiovascular Science, QMRI, Edinburgh, UK EH16 4TJ. email: r.gifford@ed.ac.uk Tel: + 44 (0) 131 2426762 Fax: + 44 (01) 131 2426779

Supplementary table 1.

Freeze-thaw cycles (n)	P4, Architect (mean (SD) % of index sample)	P4, Architect (mean (SD) % of index sample)
1	97.4 (0.93)	97.8 (6.01)
2	98.1 (2.83)	100.0 (6.70)
3	95.1 (2.99)	100.0 (7.12)
4	96.4 (0.66)	100.0 (2.43)
5	92.4 (3.23)	93.0 (2.99)

Measured P4 concentrations by freeze-thaw cycle. Male urine was spiked with 240nM P4 and underwent up to five freeze-thaw cycles. Given as percentage of index sample (zero freeze-thaw cycles).
